# Effects of essential oil coated with glycerol monolaurate on growth performance, intestinal morphology, and serum profiles in weaned piglets

**DOI:** 10.5713/ab.22.0261

**Published:** 2022-11-14

**Authors:** Yanan Wang, Juntao Li, Huakai Wang, Yihai Mi, Yongqiang Xue, Jiaan Li, Yongxi Ma

**Affiliations:** 1 State Key Laboratory of Animal Nutrition, College of Animal Science and Technology, China Agricultural University, Beijing 100193, China; 2 CALID BIOTECH (WUHAN) CO., LTD, Wuhan 430073, China

**Keywords:** Antioxidant Capacity, Essential Oil, Glycerol Monolaurate, Immunity Function, Piglets

## Abstract

**Objective:**

This study aimed to investigate the effects of essential oil coated with glycerol monolaurate (GML) on the growth performance, intestinal morphology, and serum profiles of weaned piglets.

**Methods:**

A total of 144 weaned piglets (Duroc×[Landrace×Yorkshire], average weight 8.07±3.33 kg) were randomly assigned to three groups with six replicate pens and eight piglets per pen: i) CON: a corn-soybean basal diet; ii) LEG: with 1,000 mg/kg essential oil coated with GML; and iii) HEG: with 2,000 mg/kg essential oil coated with GML.

**Results:**

Results showed that average daily gain was increased (p<0.05) linearly by essential oil coated with GML supplementation on day 14 to 28 and day 0 to 28 compared with the CON group. Dietary supplementation with HEG increased (p<0.05) total antioxidant capacity and catalase activity on day 14, and immunoglobulin A (IgA) and IgM concentration on day 28 and tended to increase IgG on day 28. In addition, the crypt depth in the jejunum was reduced (p<0.05), and villus height and villus height/crypt depth in the ileum were increased (p<0.05) in the HEG group compared with the CON group. Moreover, lower (p<0.05) concentrations of tumor necrosis factor-α, interferon-γ, interleukin-1β (IL-1β), IL-8, and IL-10 were observed in the jejunum of piglets supplemented with HEG compared with the CON group. In addition, dietary HEG tended to decrease IL-6 level in the jejunum of piglets compared with the CON group.

**Conclusion:**

Dietary essential oil coated with GML can improve growth performance of weaned piglets. Moreover, supplementing 2,000 mg/kg essential oil coated with GML was demonstrated to improve antioxidant ability, and intestinal morphology, and reduce jejunal inflammatory factor levels.

## INTRODUCTION

Weaning stress often causes a series of problems for piglets, such as a high frequency of diarrhea, oxidative damage, decreased feed intake, and poor growth performance [1]. Over the past few decades, antibiotics have been widely used to alleviate weaning stress and improve growth performance via modulating gut health in weaning piglets [2]. However, overuse of antibiotics has led to antibiotic resistance, which threatens human public safety. Antibiotic growth promoters were banned in the European Union in January 2006 due to concerns about antibiotic residues [3]. As a result, natural feed additives have become alternatives for controlling pathogens, improving animal production, and maximizing feed efficiency.

As a bioactive plant compound, essential oil (including thymol and cinnamaldehyde) has been found to have antibacterial properties [4]. Dietary thymol supplementation promoted the growth of intestinal beneficial bacteria and inhibited the growth of potentially harmful bacteria, including *Escherichia coli* and *Clostridium perfringens* in broilers [5,6]. Moreover, cinnamaldehyde can reduce egg-borne transmission of *S. Enteritidis* [7] and has the potential to improve the taste of the feed and increase the feed intake of livestock and poultry as a spice. Glycerol monolaurate (GML) is a monoglyceride composed of lauric acid and glycerol that has been approved by the US Food and Drug Administration as a Generally Recognized a Safe food emulsifier [8]. A great number of studies have shown that GML is beneficial in enhancing growth performance, meat quality, and egg quality coincident with modulating immune status and intestinal microbiota for broilers [9,10]. The previous result in our group also indicates piglets fed with diets containing GML show reduced diarrhea rate and improved intestinal morphology, antioxidant capacity, and immune status in weaned piglets [11]. Hence, we speculate that essential oil coated with GML can be an ideal feed additive for weaned piglets to improve growth performance. However, few studies have been conducted on the effects of the combination of essential oil and GML on piglets. Therefore, the objective of this study was to evaluate the effects of essential oil coated with GML on performance, antioxidant capacity, immunity status, jejunal inflammatory factor levels, and intestinal morphology in weaned piglets.

## MATERIALS AND METHODS

All animal experiments were approved by the animal ethical committee of China Agricultural University (Beijing, China; No. AW90602202-1-2). All animal experiments were performed at the FengNing Swine Research Unit of China Agricultural University (Chengdejiuyun Agricultural and Livestock Co., Ltd., Hebei, China). The main active components of the essential oil coated with GML used in the study are GML (800 g/kg), cinnamaldehyde (54 g/kg), and thymol (6 g/kg) and provided by CALID BIOTECH (WUHAN) CO., LTD (Wuhan, China).

### Animals and experimental design

A total of 144 (Duroc×[Landrace×Yorkshire]) weaned piglets with an average initial body weight (BW) of 8.07±3.33 kg were randomly allotted to three treatments with six replicates (8 piglets per replicate pen) per treatment based on BW and sex. The corn-soybean basal diets (CON) were formulated to meet the recommended requirements [12] and are shown in Table 1. The dietary treatments included a corn-soybean basal diet, CON with 1,000 mg/kg essential oil coated with GML supplementation (LEG), and CON with 2,000 mg/kg essential oil coated with GML supplementation (HEG). The piglets were housed in pens with relatively constant temperature (26°C to 28°C) and humidity (60% to 70%) and were given access to feed and water *ad libitum* throughout the experiment period (28 days).

### Data recording, sample collection, and analysis

Piglets were weighed individually on days 0, 14, and 28 to calculate average daily gain (ADG), and daily feed intake was recorded to calculate average daily feed intake (ADFI) and feed efficiency (gain to feed ratio, G:F ratio). Since the dietary essential oil coated with GML increased linearly the growth performance during the whole experimental period, we selected the HEG group to explore the possible mechanism by which the essential oil coated with GML improves the growth performance in piglets in the next analysis.

One piglet in each pen from CON and HEG groups was selected to obtain blood samples via jugular vena puncture with vacutainer tubes (Becton Dickinson Vacutainer Systems, Franklin Lakes, NJ, USA) at 0700 h on the morning of days 14 and 28. The blood samples were centrifuged at 3,000×*g* for 10 min to obtain the serum samples, then stored at −20°C until analysis. Enzyme-linked immunosorbent assay (ELISA) was performed to measure total antioxidant capacity (T-AOC), superoxide dismutase (SOD), malonaldehyde (MDA), glutathione peroxidase (GSH-Px), catalase (CAT), immunoglobulin A (IgA), immunoglobulin G (IgG), and immunoglobulin M (IgM) following the manufacturers’ instructions (Nanjing Jiancheng Bioengineering Institute, Nanjing, China). Briefly, the T-AOC was detected using a spectrophotometer (LengGuang SFZ1606017568, Shanghai, China) at 520 nm since it can be reflected by developed stable and colored chelates. The SOD activity was quantified with the xanthine superoxide anion radical product and oxidase reaction system, and the MDA concentration was quantified with the thiobarbituric acid reactive substances method. The activity of GSH-Px was quantified by measuring the consumption rate of reduced glutathione in the enzymatic reaction. The CAT activity was detected at 240 nm based on the consumption of H_2_O_2_. According to the optical density values of standards, IgA, IgG, and IgM were measured with UV-2401PC at 700 nm, 340 nm, and 340 nm, respectively (UV–vis recording spectrophotometer, SHIMADZU Corporation, Tokyo, Japan). The immunoglobulin concentration of the samples (g/L) was calculated with the standard curve.

At the end of the experimental period, one piglet in each pen from CON and HEG groups with a BW close to average was euthanized to collect intestinal samples (duodenum, jejunum, and ileum). The intestinal samples were fixed in 4% paraformaldehyde. Then, the fixed samples were cleared, dehydrated, and embedded in paraffin wax, and sectioned at 5 μm thickness and installed on glass slides, and stained with hematoxylin and eosin. Villus height at least 12 randomly orientated villi and their adjoining crypts were measured with a light microscope with 40×combined magnification using image processing and an analysis system (version 1; Leica Imaging Systems Ltd., Cambridge, UK).

The activity of tumor necrosis factor-α (TNF-α), interferon-γ (IFN-γ), interleukin-1β (IL-1β), interleukin-6 (IL-6), interleukin-8 (IL-8), and interleukin-10 (IL-10) in jejunum was measured using ELISA kits (Nanjing Jiancheng Bioengineering Institute, Nanjing, China). Following the kit manufacturer’s operating instructions, the levels of TNF-α, IFN-γ, IL-1β, IL-6, IL-8, and IL-10 were calculated with the standard curve and were expressed as nanograms per liter.

### Statistical analysis

The data were analyzed as a randomized complete block design using the general linear model procedure of SAS 9.2 (SAS Institute, Cary, NC, USA). The statistical model included the diets (n = 2) as fixed effect, and the blocks (n = 2) as random effects. Pen was used as experimental unit for the growth performance and individual pig as experimental unit for serum parameters, intestinal morphology, and jejunal inflammatory factors. Data was analyzed by using orthogonal polynomial contrast. Significance was taken at p<0.05 and tendency at 0.05<p<0.10.

## RESULTS

### Growth performance

The effects of essential oil coated with GML supplementation on growth performance in weaned piglets are shown in Table 2. Essential oil coated with GML supplementation increased (p<0.05) linearly the ADG on day 14 to 28 and day 0 to 28.

### Antioxidant capacity in serum

Table 3 shows the effects of supplementation with essential oil coated with GML on the activity of antioxidant enzymes in the serum of piglets. On day 14, higher serum T-AOC and CAT activity were observed in piglets supplemented with HEG compared with the CON group (p<0.05). However, no treatment differences were detected in the activity of SOD, MDA, and GSH-Px on day 14 nor 28 in serum.

### Immunity status in serum

As shown in Table 3, dietary HEG supplementation increased IgA and IgM concentration in the serum on day 28 (p<0.05), while did not affect IgG concentration.

### Intestinal morphology

As shown in Table 4, dietary supplementation with HEG reduced (p<0.05) crypt depth in the jejunum and increased (p<0.05) villus height and villus height to crypt depth ratio in the ileum compared with the CON group. However, HEG did not affect the morphology of the duodenum.

### Jejunal inflammatory factor

As shown in Table 5, dietary supplementation with HEG reduced the concentration of TNF-α, IFN-γ, IL-1β, IL-8, and IL-10 compared with the CON group (p<0.05).

## DISCUSSION

The antimicrobial and antioxidant properties of essential oil make it widely used as a performance enhancer in animal production [4]. Moreover, GML also is a growth-promoting additive due to its antibacterial, virucidal, fungicidal, and anti-inflammatory properties [13]. In the present study, we demonstrated that essential oil coated with GML supplementation, which contained GML (800 g/kg), cinnamaldehyde (54 g/kg), and thymol (6 g/kg), improved linearly ADG in weaned piglets compared with CON group on day 14 to 28 and day 0 to 28, and ADG increased from 8.5% to 10.3% on day 0 to 14, although there was no statistical difference. Similar results were found in the study of Snoeck et al [14] who reported GML presents a capacity to be used as a growth enhancer in replacement to the use of antibiotics in piglets. Furthermore, Liu et al [9,10] reported that dietary supplementation of GML improves the productive performance of broilers and the main reason for the improvement of growth performance is the increased ADFI by GML supplementation. However, Li et al [11] reported GML had no effect on ADG, ADFI, and G:F ratio in weaned piglets. Also, Cui et al [15] found GML did not improve the growth performance in low-protein diets in weaned piglets. The discrepancies may be due to the dosage of the GML used or the conjunctive use of GML and essential oil in our study. Furthermore, cinnamaldehyde can improve feed digestion by stimulating the secretion of salivary enzymes and pancreatic amylase [16], and thymol can improve weight gain and feed conversion by increasing the growth of non-pathogenic anaerobic and gram-positive facultative bacteria [6], which may also partly explain the improvement of growth performance in this study. Hashemipour et al [6] reported that dietary inclusion of 100 and 200 mg/kg thymol+carvacrol improved BW gain and feed conversion ratio in broilers. Pirgozliev et al [16] found that dietary inclusion of a commercial blend of phytogenic feed additives containing 3% cinnamaldehyde improved overall growth performance in broiler chickens. These results also verified the positive effect of thymol and cinnamaldehyde in animal production.

Lipid peroxidation can cause a series of metabolic disorders and reduced immune function, form a chain reaction of oxygen free radicals, damage the biofilm and its function, and cause damage to the body [17]. In the present study, the level of T-AOC was higher in HEG group, indicating that HEG could play an important role in preventing lipid peroxidation. Catalase is an endogenous antioxidant enzyme that can delay lipid and protein oxidation by scavenging superoxide radicals or degrading hydrogen peroxide [18]. Our results showed that dietary HEG supplementation had a higher serum CAT level, indicating that HEG can enhance the antioxidant capacity by increasing the antioxidant enzyme level. Consistent with our results, previous studies demonstrated that GML can increase T-AOC activity in serum in broilers and enhance the antioxidant capacity in piglets [10, 11]. The improvement in antioxidant ability in the HEG-treated piglets may be related to lowered inflammatory response and down-regulated toll-like receptors-4/nuclear factor kappa-B (TLR4/NF-κB) pathway [19]. Molania et al [20] found that cinnamaldehyde also can improve the salivary T-AOC in gamma-irradiated rats, and the related mechanism may be the inhibition of matrix metalloprotease (MMP) expression by cinnamaldehyde through the p38 MAPK pathway due to oxidative stress related to the MMP upregulation [21]. Moreover, thymol can improve sperm quality by decreasing oxidative damage in rats [22], this may be a pathway associated with the improvement of adenylate kinase activity [23]. Taken together, essential oil coated with GML could improve the antioxidant capacity, and thus be in favor of the improvement of growth performance.

The immune system is the body’s defensive structure, which can recognize and eliminate foreign bodies, foreign pathogenic microorganisms, and other factors that cause internal environment fluctuations. In the present research, HEG supplementation increased the concentrations of IgA and IgM, suggestive of a direct stimulatory effect on humoral immunity. The development of antibody-mediated immune responses is critical for piglets, which are vulnerable to external pathogenic microorganisms due to an underdeveloped immune system [24]. Previous studies demonstrated that supplementation with a plant essential oil containing 13.5% thymol and 4.5% cinnamaldehyde can increase concentrations of serum IgG and IgM in piglets [25], which was mainly due to their antimicrobial activity because the maturation and optimal development of the immune system depends on the development and composition of the indigenous microflora [26]. Additionally, Kong et al [19] reported that dietary GML can ameliorate intestinal morphology and barrier function by modulating intestinal immunity in broiler chicks. Witcher et al [27] reported that the calcium-dependent inositol phospholipid pathway, a well-characterized cell signaling pathway linking membrane effects to IL-2 regulation, may be a viable candidate as a target for GML to exert immune function. Therefore, essential oil coated with GML While the ability of essential oil coated with GML to influence the development of specific immune responses in piglets remains to be established, if confirmed, it would be highly valuable in animal production.

The small intestine is the site for digestion and absorption of nutrients in pigs. Villus height, crypt depth, and villus height to crypt depth ratio directly reflect the health of the small intestine. Higher villus height and villus height to crypt depth ratio, and lower crypt depth will increase mucosal surface area, resulting in an enhanced digestive capacity in pigs [28]. Environmental stress causes intestinal villi atrophy and crypt hyperplasia, resulting in decreased nutrient absorption capacity during the weaning stage of piglets [29]. In this study, the H&E staining results revealed that HEG supplementation increased villus height and villus height to crypt depth ratio of ileum and decreased crypt depth of jejunum, which can partially explain the improvement of ADG and G:F ratio. This finding agreed with our previous study, which showed that GML can increase villus height of jejunum and ileum and the ratio of villus height to crypt depth of duodenum [12]. Cui et al [15] found that GML can inhibit the increase of the intestinal mucosal permeability by modulating D-lactic acid (D-LACT) of serum in weaned piglets, as well, GML can effectively protect the integrity of the intestinal barrier by regulating the protein levels of claudin-1, occluding, and zonula occluden 1 (ZO-1). Moreover, Yang et al [30] reported that dietary supplementation with encapsulated cinnamaldehyde can increase nutrient digestibility by increasing villus height to crypt depth ratio of duodenum and jejunum. Dietary thymol in broilers could decrease the number of intestinal lesions by increasing the villus [31]. Thus, essential oil coated with GML ameliorated growth performance through modulating gut morphology in the current study.

Cytokines can mediate the interaction between cells and have a variety of biological functions, such as regulating immune responses and participating in the inflammatory response. In the current research, HEG reduced the concentrations of TNF-α, IFN-γ, IL-1β, and IL-8 of jejunum which was partly in agreement with the results of Chao et al [32] and Omonijo et al [33] who reported that cinnamaldehyde exerts anti-inflammatory effect by inhibiting the phosphorylation of extracellular signal-regulated kinase 1/2 and c-Jun N-terminal kinase 1/2, and thymol enhances barrier function and reduces reactive oxygen species production and pro-inflammatory cytokine gene expression in the epithelial cells during inflammation. In addition, our results were partially consistent with Mo et al [34] who observed that GML supplementation alleviated the colitis by modulating gut microbiota in C57BL/6 mice. Moreover, our results showed that GML reduced the level of anti-inflammatory cytokine IL-10, indicating that the CON group developed inflammatory responses in small intestine. The improvement of growth performance may be related to anti-inflammatory and antibacterial properties that reduce the release of macrophages producing pro-inflammatory cytokines, which cause tissue damage and increase the energy expenditure when produced in excess [35].

## CONCLUSION

This study indicates that dietary supplementation of essential oil coated with GML increases the growth performance of weaned piglets. Our results also showed that the addition of 2,000 mg/kg essential oil coated with GML increases antioxidant capacity and immune status in serum and regulates jejunal inflammatory-related cytokines and intestinal morphology, which favors improvement of growth performance. Therefore, essential oil coated with GML could improve the health status of weaned piglets and is a good feed additive for piglets.

## Figures and Tables

**Table 1:** Ingredient and chemical composition of the basal diets (as-fed basis, %)

Ingredients	Period 1 (day 0 to 14)	Period 2 (day 14 to 28)
Ground corn	65.23	70.15
Soybean meal, 43.82%	10	12
Soy protein isolate	5	3
Soy protein concentrate	3	2
Whey powder	8	5
Fish meal	4	4
Soybean oil	1.5	1
Dicalcium phosphate	0.82	0.42
Limestone	0.85	0.9
Salt	0.3	0.3
L-Lys HCI, 78%	0.5	0.47
DL-Methionine, 98%	0.09	0.07
L-Threonine, 98%	0.17	0.15
L-Tryptophan, 98%	0.04	0.04
Premix^1)^	0.5	0.5
Calculated nutritional levels^2)^		
Digestible energy (kcal/kg)	3,548.68	3,522.94
Crude protein (%)	20.09	18.62
SID Lysine (%)	1.35	1.23
SID^3)^ Methionine (%)	0.39	0.36
SID Threonine (%)	0.79	0.73
SID Tryptophan (%)	0.22	0.2
Calcium (%)	0.8	0.7
Available phosphorus (%)	0.4	0.33

1) The premix provided the following per kilogram of diet: vitamin A, 12,000 IU; vitamin D_3_, 2,000 IU; vitamin E, 30 IU; vitamin K_3_, 3 mg; vitamin B_1_, 3 mg; vitamin B_2_, 10 mg; vitamin B_6_, 6 mg; vitamin B_12_, 24 μg; nicotinic acid, 30 mg; D-pantothenic acid, 30 mg; folic acid, 2 mg; biotin, 0.3 mg; choline chloride, 600 mg; Fe, 120 mg; Cu, 10 mg; Mn, 35 mg; Zn, 120 mg; I, 0.3 mg; Se, 0.3 mg.

2) Nutrient levels were calculated according to NRC [12].

3) SID, standardized ileal digestible.

**Table 2:** Effects of dietary essential oil coated with glycerol monolaurate (GML) on growth performance in piglets

Item	Treatment^1)^			SEM	p-value	
	
	CON	LEG	HEG		Linear	Quadratic
Body wt., 0 d (kg)	8.00	8.03	8.17	0.15	0.65	0.86
Body wt., 14 d (kg)	11.82	12.17	12.38	0.22	0.31	0.88
Body wt., 28 d (kg)	16.89	18.04	18.17	0.30	0.09	0.42
day 0 to 14						
ADG (g/d)	272	296	301	8.14	0.16	0.60
ADFI (g/d)	484	527	492	23.73	0.89	0.47
G:F ratio	0.57	0.56	0.63	0.03	0.26	0.26
day 14 to 28						
ADG (g/d)	363	420	414	9.07	<0.05	0.09
ADFI (g/d)	701	803	711	31.73	0.90	0.17
G:F ratio	0.54	0.53	0.59	0.06	0.28	0.29
day 0 to 28						
ADG (g/d)	318	358	357	7.63	<0.05	0.20
ADFI (g/d)	592	665	601	27.06	0.90	0.27
G:F ratio	0.56	0.54	0.60	0.04	0.19	0.19

SEM, standard error of the mean (n = 6); BW, body weight; ADG, average daily gain; ADFI, average daily feed intake; G:F ratio, gain to feed ratio.

1) CON, piglets in the control group fed a basal diet; LEG, with 1,000 mg/kg essential oil coated with GML supplementation; HEG, with 2,000 mg/kg essential oil coated with GML supplementation.

**Table 3:** Effects of dietary essential oil coated with glycerol monolaurate (GML) on serum antioxidant capacity and immunity status in piglets

Items	Treatment^1)^		SEM	p-value

	CON	HEG		
day 14				
T-AOC (U/mL)	8.55	9.65	0.28	<0.05
SOD (U/mL)	52.87	55.27	0.87	0.12
MDA (nmol/mL)	1.52	1.53	0.03	0.88
GSH-Px (umol/L)	8.64	7.71	0.52	0.41
CAT (U/mL)	10.04	13.85	0.73	<0.05
IgA (μg/mL)	19.12	20.84	0.73	0.26
IgG (mg/mL)	10.08	10.14	0.32	0.93
IgM (μg/mL)	8.10	8.29	0.15	0.56
day 28				
T-AOC (U/mL)	9.33	9.65	0.16	0.34
SOD (U/mL)	51.06	52.16	0.88	0.56
MDA (nmol/mL)	1.60	1.67	0.03	0.22
GSH-Px (umol/L)	14.86	13.37	0.62	0.25
CAT (U/mL)	13.76	14.34	0.51	0.27
IgA (μg/mL)	15.51	19.06	0.73	<0.05
IgG (mg/mL)	9.44	10.20	0.23	0.10
IgM (μg/mL)	6.78	7.68	0.20	<0.05

SEM, standard error of the mean (n = 6); T-AOC, total antioxidant capacity; SOD, superoxide dismutase; MDA, malondialdehyde; GSH-Px, glutathione peroxidase; CAT, catalase; IgA, immunoglobulin A; IgG, immunoglobulin G; IgM, immunoglobulin M.

1) CON, piglets in the control group fed a basal diet; HEG, with 2,000 mg/kg essential oil coated with GML supplementation.

**Table 4:** Effects of dietary essential oil coated with glycerol monolaurate (GML) on small intestinal morphology in piglets

Items	Treatment^1)^		SEM	p-value

	CON	HEG		
Duodenum				
Villus height (μm)	341.04	360.15	20.34	0.66
Crypt depth (μm)	558.81	614.24	27.86	0.30
Villus height/crypt depth	0.62	0.59	0.04	0.75
Jejunum				
Villus height (μm)	327.70	301.57	15.91	0.39
Crypt depth (μm)	362.82	318.27	10.92	<0.05
Villus height/crypt depth	0.91	0.96	0.05	0.55
Ileum				
Villus height (μm)	356.35	417.13	17.97	<0.05
Crypt depth (μm)	357.91	342.90	12.10	0.52
Villus height/crypt depth	1.01	1.21	0.06	<0.05

SEM, standard error of the mean (n = 6).

1) CON, piglets in the control group fed a basal diet; HEG, with 2,000 mg/kg essential oil coated with GML supplementation.

**Table 5:** Effects of dietary essential oil coated with glycerol monolaurate (GML) on jejunal inflammatory factor levels in piglets

Items	Treatment^1)^		SEM	p-value

	CON	HEG		
TNF-α (ng/g)	65.36	49.46	3.65	<0.05
IFN-γ (pg/mg)	144.57	132.47	2.65	<0.05
IL-1β (ng/g)	114.52	88.31	5.58	<0.05
IL-6 (ng/g)	40.05	30.89	2.73	0.09
IL-8 (ng/g)	100.58	70.94	5.29	<0.05
IL-10 (ng/g)	25.00	18.24	1.47	<0.05

SEM, standard error of the mean (n = 6); TNF-α, tumor necrosis factor-α; IFN-γ, interferon-γ; IL-1β, interleukin-1β; IL-6, interleukin-6; IL-8, interleukin-8; IL-10, interleukin-10.

1) CON, piglets in the control group fed a basal diet; HEG, with 2,000 mg/kg essential oil coated with GML supplementation.
